# Observation of an intermediate state during lithium intercalation of twisted bilayer MoS_2_

**DOI:** 10.1038/s41467-022-30516-z

**Published:** 2022-05-30

**Authors:** Yecun Wu, Jingyang Wang, Yanbin Li, Jiawei Zhou, Bai Yang Wang, Ankun Yang, Lin-Wang Wang, Harold Y. Hwang, Yi Cui

**Affiliations:** 1 Stanford Institute for Materials and Energy Sciences, SLAC National Accelerator Laboratory, Menlo Park, CA USA; 2 Department of Electrical Engineering, Stanford University, Stanford, CA USA; 3 Department of Materials Science and Engineering, Stanford University, Stanford, CA USA; 4 Materials Sciences Division, Lawrence Berkeley Laboratory, Berkeley, CA USA; 5 Department of Physics, Stanford University, Stanford, CA USA; 6 Department of Applied Physics, Stanford University, Stanford, CA USA

**Keywords:** Two-dimensional materials, Phase transitions and critical phenomena

## Abstract

Lithium intercalation of MoS_2_ is generally believed to introduce a phase transition from H phase (semiconducting) to T phase (metallic). However, during the intercalation process, a spatially sharp boundary is usually formed between the fully intercalated T phase MoS_2_ and non-intercalated H phase MoS_2_. The intermediate state, *i.e*., lightly intercalated H phase MoS_2_ without a phase transition, is difficult to investigate by optical-microscope-based spectroscopy due to the narrow size. Here, we report the stabilization of the intermediate state across the whole flake of twisted bilayer MoS_2_. The twisted bilayer system allows the lithium to intercalate from the top surface and enables fast Li-ion diffusion by the reduced interlayer interaction. The *E*_2g_ Raman mode of the intermediate state shows a peak splitting behavior. Our simulation results indicate that the intermediate state is stabilized by lithium-induced symmetry breaking of the H phase MoS_2_. Our results provide an insight into the non-uniform intercalation during battery charging and discharging, and also open a new opportunity to modulate the properties of twisted 2D systems with guest species doping in the Moiré structures.

## Abstract (web summary)

Li intercalation of MoS_2_ induces a transition from the insulating H-phase to the metallic T-phase, with a sharp boundary in between. Here the authors stabilize the intermediate phase in twisted bilayer MoS_2_, by leveraging the Moiré potential which facilitates fast Li diffusion and uniform intercalation.

## Introduction

Molybdenum disulfide (MoS_2_), as one of the most well-known layered transition metal dichalcogenides (TMDs) with stable semiconducting H phase and metastable metallic T phase^1–3^, has attracted intensive research interest^4^. Considerable effort has been devoted to studying the H phase MoS_2_ in the fields of electronics, photonics, etc^5,6^. The T phase has a wide variety of applications such as catalysis, energy storage, superconductivity, etc^7–10^, but the metastable nature makes it difficult to synthesis and store^1^. Therefore, various methods have been developed to induce the H to T phase transition in MoS_2_^11,12^, and these methods have flourished with the advances in atomically-thin 2D materials in recent decades^13–17^. Among these methods, lithium intercalation has been one of the most popular approaches^18,19^. Since 1983, it was found that the phase transition occurs in Li_x_MoS_2_ when 0.2 < *x* < 1^11,12^. Until now, both electrochemical and chemical methods have been developed for lithium intercalation of MoS_2_^20,21^. Electrochemical methods are widely used to provide dynamical and reversible control of the lithium intercalation of MoS_2_^11^. Chemical intercalation provides a simple approach, by soaking the MoS_2_ into highly reducible lithium solution (e.g., n-Butyllithium), where the lithium atoms can spontaneously move into the van der Waals gaps of the MoS_2_^22^. It was generally believed that lithium diffuses into MoS_2_ through the edge^13,14^. Recently, it was discovered that the lithium can also can go through across the layers via defects in thin flakes^15^.

**Fig. 1:**
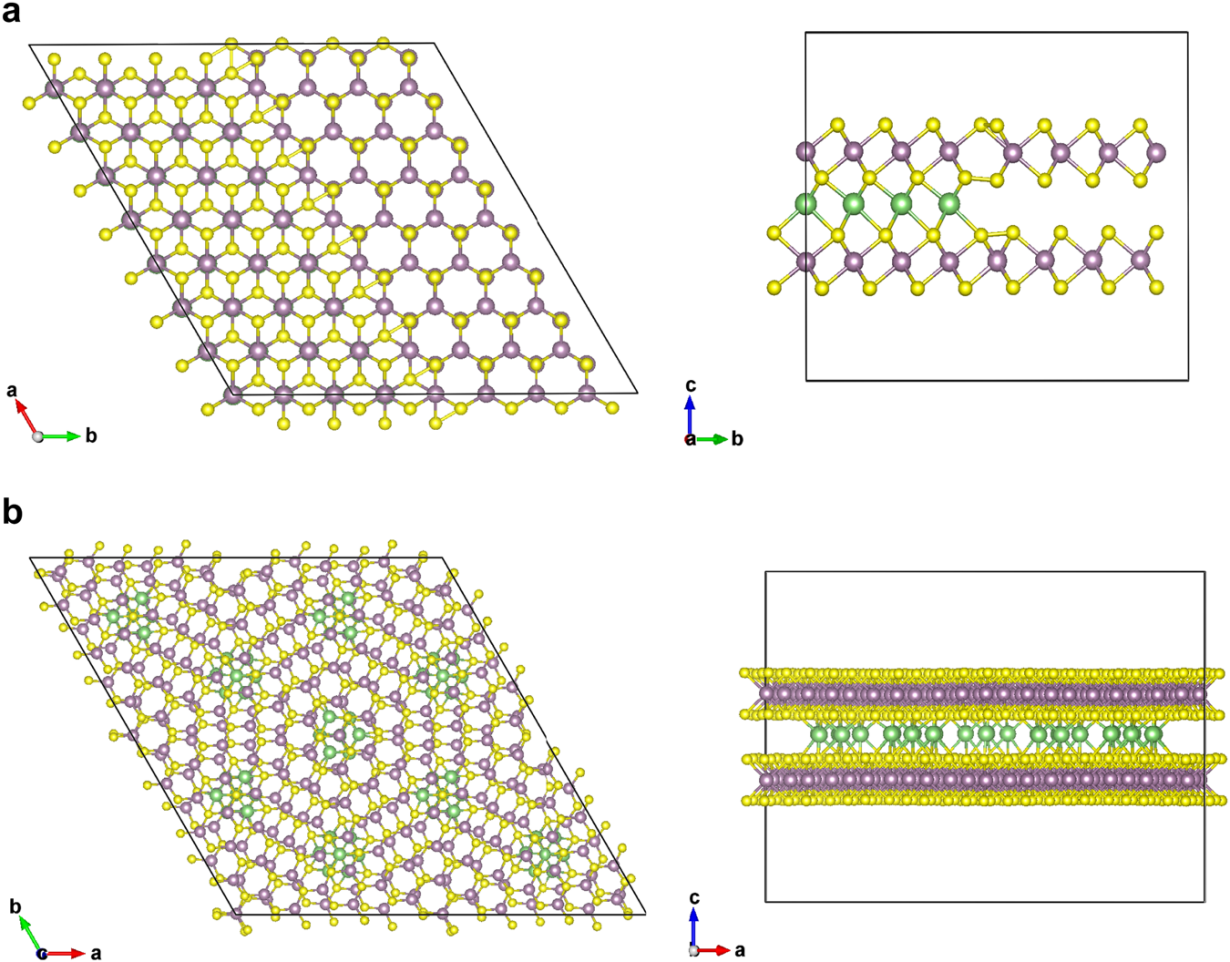
Schematic illustration of lithium intercalation in MoS_2_. **a** Lithium intercalation in bilayer 2H-MoS_2_ through the edge, resulting a phase separation (left side: top view (view along the c-axis), right side: cross-section view). **b** Lithium intercalation in twisted bilayer MoS_2_ (left side: top view (view along the c-axis), right side: cross-section view).

In recent years, a number of investigations have been performed with state-of-art characterization techniques (spectroscopic methods in particular) to study lithium intercalation of MoS_2_^13,14,23–25^. The in-situ observation of dynamic lithium intercalation into MoS_2_ under potential control by using differential optical microscopy, discovered that the intercalation starts from the edge, and distinct phase separation of lithiated and delithiated regions is observed^14^. Similar with the electrochemical intercalation, chemical intercalation also shows a distinct phase separation with a very narrow boundary region (~few hundreds of nanometers) (Fig. 1a and Supplementary Fig. 1)^13,14,23^. The intermediate state, with lithium intercalation in H phase MoS_2_ before phase transformation, is only a few hundreds of nanometers wide, which is barely studied by optical-microscopy-based techniques due to the narrow size.

The lithium diffusion in MoS_2_ is an interplay between charge-transfer process at the step and the force that pushes a row of Li ions into the interior of the basal plane of the crystal^14^. Thus, the sharp domain boundaries never show a gradual decrease in contrast with time^14^. The bilayer system provides a platform for uniform intercalation from the top surface, and the reduced interlayer interaction in the Moiré structure allows a fast diffusion of the lithium ions. The fast ion diffusion in Moiré structure originates from the reduced interlayer binding forces^26^. Therefore, instead of the formation of distinct phase boundary, the intermediate stated can be introduced in a whole flake (Fig. 1b). Here, by using twisted bilayer MoS_2_, the intermediate state can be induced in an entire flake and was signaled by a splitting of the *E*_2g_ mode in Raman spectroscopy. Our discovery offers new insight into the diffusion problem in batteries^27,28^, and also offers new possibilities to twistronics by introducing guest species in the Moiré superlattice^29,30^.

## Results and discussion

**Fig. 2:**
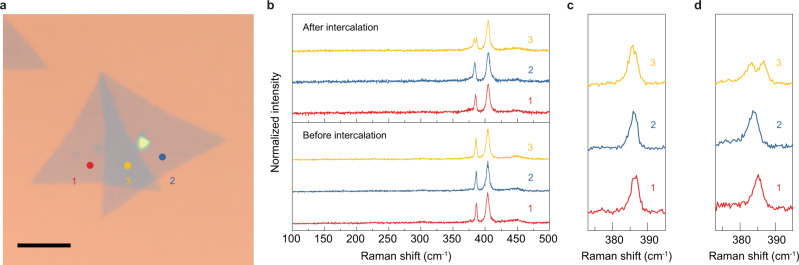
The intermediate state in lithium intercalated twisted bilayer MoS_2_. **a** Optical microscope image of a twisted bilayer MoS_2_ (scale bar 10 μm). **b** Raman spectra of the positions 1, 2, and 3 in **a** before intercalation. **c** Raman spectra of the same positions after intercalation. **d** Zoom-in image of the *E*_2g_ and *A*_1g_ peaks after intercalation.

The twisted bilayer MoS_2_ was fabricated by transferring one chemical vapor deposition (CVD) grown monolayer MoS_2_ onto another one. The transfer process was performed by PMMA- and PDMS- assisted target transfer (Supplementary Fig. 2)^31^. The twisting angle can be identified by the edge of the triangular CVD grown MoS_2_. Before intercalation, Raman spectroscopy was conducted on three different positions (Fig. 2a). Position 1 and 2 are monolayer regions on the bottom and top monolayer flakes, respectively, and position 3 is a twisted bilayer region (negligible variations of the Raman spectra in the same region were observed). As shown in the Raman spectra (Fig. 2b), before intercalation, the *E*_2g_ peak was located at ~386.1 cm^−1^ and the *A*_1g_ peak was at ~404.3 cm^−1^ in both of the monolayer regions. The ~18 cm^−1^ distance between the two modes reflected the monolayer H phase^32^. In the bilayer region, the *E*_2g_ peak showed a slight blueshift to ~385.5 cm^−1^ and the position of the *A*_1g_ peak showed negligible change. The twisting angle of 32° implies a relatively narrow *E*_2g_ and *A*_1g_ mode frequency difference in the bilayer region^33^. Twisted bilayer MoS_2_ flakes with various angles can be obtained in one transfer process (Supplementary Fig. 3).

Chemical intercalation was then conducted on the bilayer system by soaking the samples in dilute n-Butyllithium solution. After intercalation, no strong color change was observed under the optical microscope. Raman spectroscopy was performed again on positions 1, 2, and 3. It can be seen that after exposure to n-Butyllithium, both of the *E*_2g_ modes in monolayer region positions 1 and 2 shifted to smaller wavenumber. The shift of the *E*_2g_ mode resulted from the charge transfer between lithium and monolayer MoS_2_. The *E*_2g_ mode in the top layer showed a larger shift (~384 cm^−1^) than that in the bottom layer (~385 cm^−1^), which is probably due to more defects in the transferred top layer which induced more lithium absorption. The difference of the *E*_2g_ peak shift in the top and bottom monolayer region can be reduced by transferring two monolayers to ensure that every layer experiences the same transfer process (Supplementary Fig. 4). The frequencies of the *A*_1g_ mode in all three regions show negligible change after intercalation due to the low concentration of lithium during intercalation. Remarkably, the *E*_2g_ mode in the twisted bilayer region showed a striking splitting behavior (intermediate state): the original *E*_2g_ peak at ~385.5 cm^−1^ was split into two peaks at ~383 cm^−1^ and 386.6 cm^−1^ respectively. Although the *E*_2g_ modes showed slightly different shifts in positions 1 and 2, it is clear that the two split peaks in region 3 were not a simple combination of the two monolayer peaks due to the differences in frequency.

**Fig. 3:**
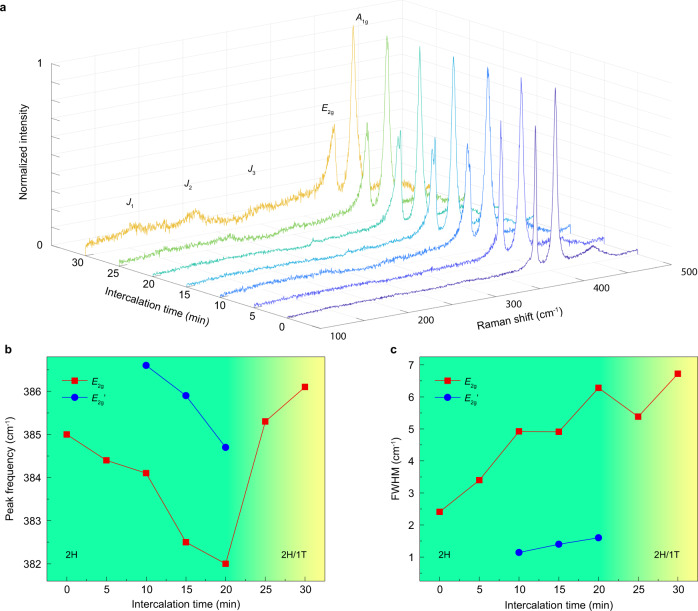
Raman spectra of the twisted bilayer MoS_2_ during intercalation. **a** Raman spectra of one twisted bilayer MoS_2_ for different intercalation times. The variation of the frequency (**b**) and FWHM (**c**) of *E*_2g_ and split *E*_2g_’ modes as a function of the intercalation time.

To reveal the Raman spectra evolution during intercalation, we immersed a twisted bilayer MoS_2_ in the n-butyllithium solution for different lengths of time (Fig. 3) and examined the Raman spectra variations. During the whole intercalation process, both the *E*_2g_ and *A*_1g_ peak were softened. As we began the lithium intercalation, the *E*_2g_ peak is slightly broadened in the first 5 min. After 10 min, the relative intensity of the *E*_2g_ mode was softened and the onset of the peak splitting was observed. More intercalation time resulted in a clear splitting and broadening of the *E*_2g_ mode. After 20 min of intercalation, we started to see the *J* modes (*J*_1_, *J*_2_, and *J*_3_) in T phase MoS_2_ and the peak splitting of the *E*_2g_ mode disappeared. Finally, after 30 min, the *J* modes were clear, indicating a complete phase transition from H to T of the intercalated MoS_2_.

The dependence of the frequencies of the *E*_2g_ peak and the split peak (which we designate as *E*_2g_’ here) on intercalation time were plotted in Fig. 3b. The peak splitting was observed after 5 min of intercalation and both the *E*_2g_ and *E*_2g_’ peak shifted to low frequency with the increase of intercalation time. The split *E*_2g_’ peak suddenly vanished when the phase transited to 1 T, suggesting that the peak splitting was only in the H phase MoS_2_. After the formation of 1 T phase MoS_2_, the *E*_2g_ peak started to shift to a higher frequency with a higher concentration of intercalated lithium. The value of the full-width-at-half-maximum (FWHM) of *E*_2g_ and *E*_2g_’ were extracted from the spectra (Fig. 3c). In general, intercalation induced a broad shear mode, which could be caused by the increased electron–phonon coupling. Around the phase transition time, the FWHM of *E*_2g_ peak showed some variations, likely caused by disorder during the phase transition.

**Fig. 4:**
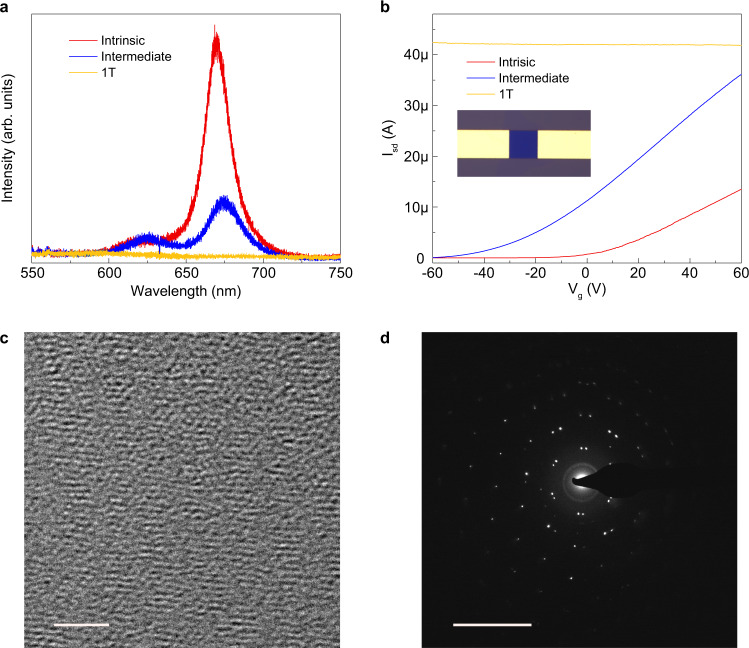
Characterizations of the intermediate state. **a** Photoluminescence of a twisted bilayer MoS_2_ sample before intercalation, in the intermediate state, and after transformation to the 1 T phase. **b** Room-temperature transfer characteristics for a twisted bilayer MoS_2_ FET before intercalation, in the intermediate state, and after transformed to 1 T phase with 1 V applied bias voltage (the width of the contact is 10 μm). **c** High resolution cryo-TEM image (scale bar: 2 nm) of a twisted bilayer MoS_2_ after 15 min lithium intercalation and **d** the corresponding electron diffraction pattern (scale bar: 10 nm^−1^).

To further characterize the samples in the intermediate state, photoluminescence (PL) was conducted on a twisted bilayer MoS_2_ in intrinsic, intermediate, and 1 T states (Fig. 4a). The PL spectra beyond 750 nm of another sample are shown in Supplementary Fig. 5, where an indirect bandgap peak can be observed^34^. The ratio of A and B excitons and the position of the indirect bandgap peak of the twisted bilayer MoS_2_ are angle-dependent, which is related to the interlayer coupling^31,34^. The location of A excitons was at ~670 nm, indicating the bandgap of the twisted bilayer MoS_2_ was ~1.85 eV. For the intermediate state, the intensity of the PL became much weaker with a redshift of the A exciton, signifying a narrower bandgap, but also indicating that the MoS_2_ still maintained the semiconducting H phase. It should be noted that some lithium residue on the surface of MoS_2_ could affect the PL intensity, but the H phase was not affected by a degree of surface doping^35^. Another twisted bilayer MoS_2_ sample was fabricated as a field-effect transistor to study the variation of conductivity (Fig. 4b insert). By sweeping the gate voltage from −60 V to 60 V, the source-drain current was measured with a constant bias voltage of 1 V. Before intercalation, the device showed typical *n*-type semiconductor behavior. For the intermediate state, the sample still maintained *n*-type behavior with one order of magnitude enhancement of the conductivity at zero gate voltage. The fully intercalated T phase twisted bilayer MoS_2_ showed a constant conductivity regardless of the gate voltage (additional electrical characterization at different source-drain voltages can be seen in Supplementary Fig. 6). Cryogenic transmission electron microscopy (TEM) was used to characterize the twisted bilayer samples after 15 min of intercalation. The Moiré pattern was observed under high-resolution TEM and confirmed by the two series of MoS_2_ lattices with a twist angle in the electron diffraction pattern. However, given both the low concentration of intercalated lithium and relatively small atomic number of lithium, the lithium was not directly observable in the TEM. Nevertheless, electron energy-loss spectroscopy (EELS) shows that lithium is incorporated in the bilayer system (Supplementary Fig. 7).

The splitting of the shear mode was observed in lithium intercalation of graphite but never seen in MoS_2_^36,37^. In graphite, the intercalants shift the frequency of the shear mode, and incomplete intercalation induced a mixture of bare and intercalated graphite, which results in the observed double peak^30^. However, the situation is different with our twisted bilayer MoS_2_ case since we have uniform lithium intercalation in the bilayer system without a staging effect. Recent calculations showed that in twisted bilayer systems, some high-symmetry points exist in the Moiré superlattice that is energy favorable for lithium^38,39^. When lithium goes into the twist bilayer MoS_2_, it tends to fill these specific regions of the Moiré superlattice and then distribute to other equivalent locations. To understand the physics behind these phenomenon, first-principles calculations were conducted based on density function theory (DFT) to simulate the phonon spectrum.

Since the intermediate state was observed at different angles, we started from a twisted bilayer Moiré supercell with a random twisting angle ((3,4)-configuration with a twist angle of 9.43°, Supplementary Fig. 8a). To study the effect of Li intercalation in twisted bilayer MoS_2_, we constructed two additional models where one Li atom is placed in between the twisted bilayers and the stacking order locally is approximately MX (Supplementary Fig. 8b) and MM (Supplementary Fig. 8c) respectively. MX (MM) refers to the stacking order where the metal atom in the top MoS_2_ layer is directly on top of the chalcogenide (metal) atom in the bottom MoS_2_ layer^40^. The MX and MM sites locally possess approximate hexagonal symmetry as in the untwisted MoS_2_ bilayer. The Li atom is placed at a tetrahedrally coordinated site bonded with four S atoms. The phonon density of states (PDOS) of the pristine twisted bilayer MoS_2_ and the two Li-intercalated configurations were calculated and plotted in Supplementary Fig. 8d–f. The PDOS for the Li-intercalated twisted bilayer MoS_2_ showed a significant broadening across the frequency spectrum compared with that of the pristine twisted bilayer MoS_2_. This indicates that the presence of the Li atom reduces the symmetry of the lattice locally, increasing the anharmonicity of vibrational eigenmodes of the whole lattice. On the other hand, due to the very low Li concentration in these configurations (<2%), the frequencies of the eigenmodes of MoS_2_ vibrations in the Li-intercalated models do not differ noticeably compared to the pristine MoS_2_ case.

**Fig. 5:**
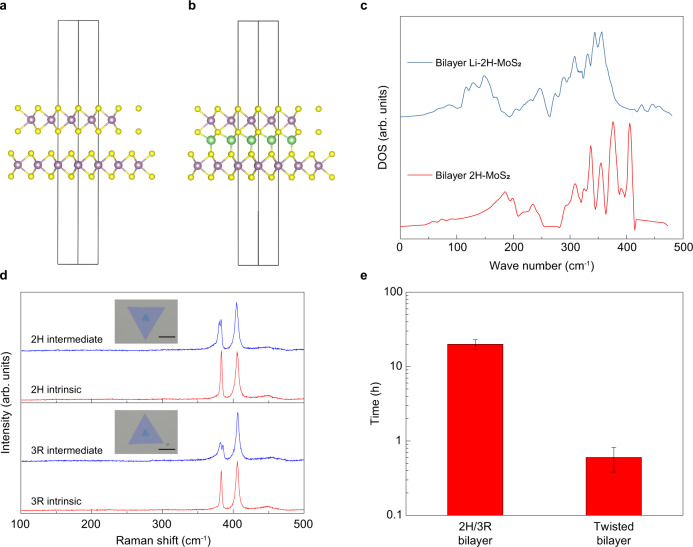
The intermediate state of untwisted bilayers. Schematic illustration of bilayer 2H-MoS_2_ (**a**) and fully intercalated bilayer 2H-MoS_2_ (**b**). **c** The calculated phonon DOS of the structure in **a** and **b. d** CVD grown bilayer 2H and 3R MoS_2_, and the corresponding Raman spectra in the intrinsic and intermediate state (scale bar: 10 μm). **e** The intercalation time required for phase transition of CVD grown 2H/3R bilayer MoS_2_ and transferred twisted bilayer MoS_2_. The error bars are standard deviations based on three samples for each condition.

However, it is challenging to predict the phonon dispersion with more lithium in the Moiré supercell, because the variations of lithium positions and phonon DOSs are exponentially sensitive to the amount of lithium in the system. Therefore, we simulate an extreme case: 180° rotation, i.e., 2H bilayer MoS_2_ (Fig. 5a). Usually, when the H phase MoS_2_ is fully lithiated, a phase transition from H to T is unavoidable, yet here we consider the structure of the H phase so that the effect of lithium intercalation in the H phase MoS_2_ can be examined. The phonon DOS of pure bilayer 2H-MoS_2_ was calculated and shown by the blue curve in Fig. 5c. The calculated *E*_2g_ and *A*_1g_ modes were located at ~374 cm^−1^ and 406 cm^−1^ respectively. The *A*_1g_ mode is singly degenerate and the *E*_2g_ mode is doubly degenerate due to the lattice symmetry. For fully intercalated 2H MoS_2_, each lithium atom occupies a tetrahedrally coordinated site bonded with four S atoms, where the local stacking order is MX (Fig. 5b). In the DOS of the fully lithiated 2H MoS_2_, the *E*_2g_ mode was split into two peaks located at 341 cm^−1^ and 353 cm^−1^ with a large shift and the broadening. In fact, the curves in Fig. 5c were smoothed from the raw data in Supplementary Fig. 9, and the two split peaks originated from a combination of multiple modes. The symmetry-breaking caused by lithium in the H phase MoS_2_ induced the formation of multiple modes. The simulated atomic vibrational movements associated with the *E*_2g_ mode before and after intercalation is shown in the Supplementary Movie 1. In Li-MoS_2_, the lower frequency in-plane modes correspond to the vibration of the upper layer MoS_2_, and higher frequency in-plane modes corresponds to the bottom layer vibration. The *A*_1g_ mode was extinguished in the fully lithiated 2H-MoS_2_ due to the dissociation of interlayer bonding due to lithium, which is similar with the 1 T MoS_2_ with lithium^13^. Consequently, we believe that the splitting of the *E*_2g_ mode was caused by the lithium-induced symmetry breaking in MoS_2_. The twist structure is not thermodynamically essential for the peaking splitting behavior. Hence, we should expect the peaking splitting behavior in non-twisted bilayer MoS_2_.

The intermediate state was observed in both 2H and 3R MoS_2_ (Fig. 5d). However, since the Li ion diffusion in the CVD grown bilayers is much slower than that in the twisted bilayer MoS_2_, the time required for the observation of CVD bilayers shows large variations, and a sharp 2H/1T boundary can be observed in some samples due to the large amount of lithium intercalation through the edges. To qualitatively evaluate the ion diffusivity in MoS_2_, we summarized the time required for observing the phase transition in untwisted bilayers and twisted bilayers as shown in Fig. 5e. The intercalation time for the phase transition of the untwisted bilayer is over one order of magnitude longer than that of the twisted bilayer, suggesting a faster ion diffusion in the twisted bilayers. The peak splitting behavior can also be induced in the exfoliated bilayer MoS_2_ (see Supplementary Fig. 10). Therefore, although the twist structure is not thermodynamically necessary for the formation of peaking splitting, it facilities the fast kinetics of lithium diffusion, enabling an accessible observation of the phenomenon.

The intercalation time of the twisted bilayer MoS_2_ showed some variations, which is probably due to the variations of the twist angle. The twist angle not only defines the density of the localized energy favorable position^38^, but also controls the interlayer binding energy. A recent study shows that the ion diffusion in van der Waals interlayer is strongly related to the van der Waals interlayer expandability, and a higher interlayer expandability usually leads to a higher ion diffusivity^26^. Previous reports have demonstrated the angle dependence of the interlayer coupling of twisted bilayer MoS_2_^31,34^, suggesting an angle dependence of the ion diffusion in twisted bilayer MoS_2_. However, due to the low controllability of chemical intercalation and the uncertainty of the measurement of ion diffusivity by optical methods, further efforts are needed to accurately investigate the dependence of twist angle and the kinetics of ion diffusion.

In conclusion, we have observed an intermediate state during lithium intercalation of twisted bilayer MoS_2_ by Raman spectroscopy. The bilayer structure allows lithium to intercalate from the top surface and the Moiré pattern facilitates the spread of lithium in MoS_2_. The splitting of the shear mode was caused by the lithium-induced symmetry breaking in MoS_2_. Our discovery is meaningful for understanding nonuniform intercalation in battery materials during fast charge and discharge^41^, and presents a potential spatially distributed doping technique for twistronics^42^.

## Methods

### Synthesis of Monolayer of MoS_2_

Monolayer MoS_2_ was synthesized by chemical evaporation deposition (CVD) on 300 nm SiO_2_/Si substrate with molybdenum oxide (MoO_3_) and sulfur as precursors, and argon as the carrier gas, at a growth temperature of 750 °C^43^. Sodium chloride (NaCl) can be used in the growth to improve the yield of monolayer growth^44^. The detailed transfer process is described in the Supplementary Note 1.

### Chemical intercalation

The chemical intercalation was conducted by immersing the samples into 1.6 M n-Bbutyllithium hexane solution at room temperature in an argon-filled glovebox with oxygen and water concentration less than 0.1 ppm. After intercalation, samples were washed by anhydrous hexane for several times and dried by argon gas flow to remove organic residues. The intercalated samples were sealed in the argon glove box before transferring out for other characterizations.

### Characterization

The Raman data was obtained by using a Horiba Labram HR Evolution Raman System with 532 nm laser wavelength and 1800 l/mm grading. The ideal resolution is ~1/3 cm^−1^ in the measurement range. The ideal laser spot size was 564 nm in diameter under a 100× objective. The FET devices were fabricated by e-beam lithography and e-beam evaporation of 3/50 nm Cr/Au to form contacts. The electrical tests were conducted on an Agilent B1500A Semiconductor Device Parameter Analyzer. Cryo-TEM experiments were carried out using a FEI Titan 80-300 aberration-corrected environmental TEM operated at 300 kV with Gatan 626 cryo-transfer holder. The TEM sample was prepared by first transferring the bilayers onto TEM grid, and then conducting intercalation. The TEM grid was transferred from glovebox to the TEM holder by using the cryo-transfer method^45^.

### Density Functional Theory Calculations

The calculations were performed with the Density Functional Theory (DFT) code Quantum ESPRESSO^46^. The non-local functional vdW-df-cx^47^ is used as the exchange-correlation functional to describe the interlayer van der Waals interaction. The Projected Augmented Wave (PAW) pseudopotentials of PSlibrary1.0^48^ are used to describe the effective core electron wavefunctions of Mo, S, and Li. The 7 × 7 × 1 k-point grid and the Gamma k-point are used for the (3,4) twisted MoS_2_ bilayer supercells and the 2H-MoS_2_ unit cells, respectively. The energy cutoff is chosen to be 680 eV. The energy and forces are converged to within 10^−4^ eV and 10^−3^ eV/Å respectively. The length of the vacuum is 15 Å to minimize artificial interlayer interaction due to periodicity of supercell. The phonon calculations are performed using Density Functional Perturbation Theory (DFPT)^49^ for the 2H-MoS_2_ unit cells, and finite displacement method for the (3,4) twisted MoS_2_ bilayer supercells. The 5 × 5 × 1 q-point grid is used for the DFPT calculations; the length of the displacement is 0.02 Å.

## Supplementary information

Supplementary Information

Description of Additional Supplementary Files

Supplementary Movie 1

## Data Availability

The data that support the findings of this study are available from the authors upon reasonable request.

## Code availability

Codes related to the DFT calculations are available from the authors upon reasonable request.
