# Supplementary material for: Observation of an intermediate state during lithium intercalation of twisted bilayer MoS2
Source: Nat Commun. 2022 May 30;13:3008. doi: 10.1038/s41467-022-30516-z (PMC9151788; doi:10.1038/s41467-022-30516-z)
Supplement: Supplementary file 1 — Supplementary Information [file 41467_2022_30516_MOESM1_ESM.pdf]

Supplementary Information for

Observation of an intermediate state during lithium intercalation of twisted bilayer MoS_2

Yecun Wu[1,2]*, Jingyang Wang[3,4]*, Yanbin Li[3], Jiawei Zhou[3], Bai Yang Wang[1,5], Ankun Yang[3], Lin-Wang Wang[4], Harold Y. Hwang[1,6†], Yi Cui[1,3†]

1. Stanford Institute for Materials and Energy Sciences, SLAC National Accelerator Laboratory, Menlo Park, CA, USA.

2. Department of Electrical Engineering, Stanford University, Stanford, CA, USA.

3. Department of Materials Science and Engineering, Stanford University, Stanford, CA, USA.

4. Materials Sciences Division, Lawrence Berkeley Laboratory, Berkeley, CA, USA.

5. Department of Physics, Stanford University, Stanford, CA, USA.

6. Department of Applied Physics, Stanford University, Stanford, CA, USA.

†Corresponding authors. Email: hyhwang@stanford.edu (H.Y.H.); yicui@stanford.edu (Y.C.);

*These authors contributed equally to this work.

This Supplementary Information document includes:

Supplementary Note 1

Supplementary Figures 1 - 10.

Supplementary References

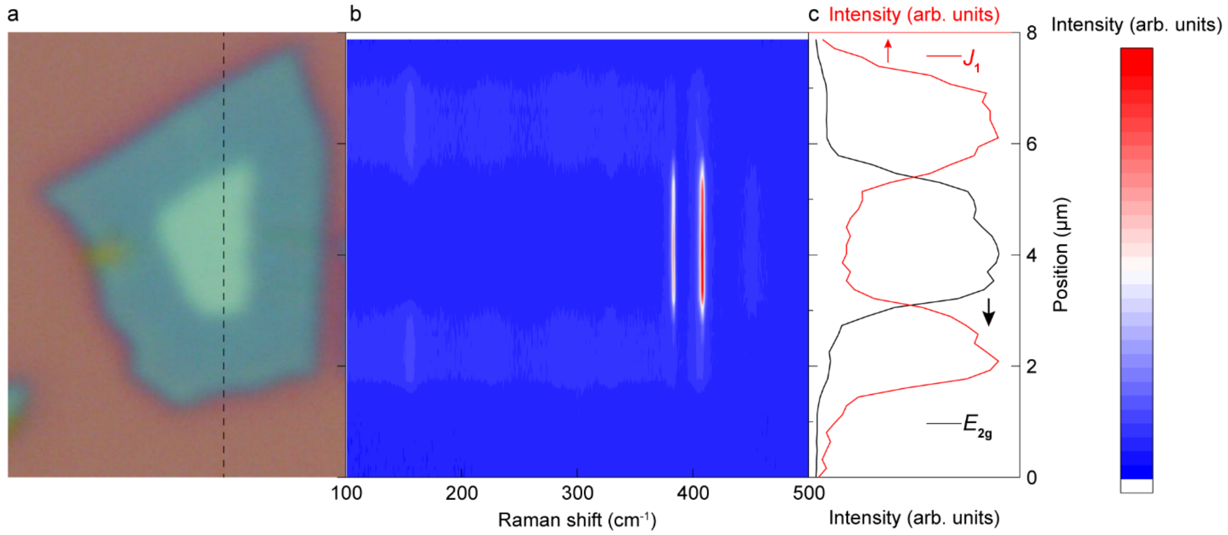

Supplementary Figure 1. a, Optical microscope image of the exfoliated few layer MoS_2. The dash line indicates the 50-point Raman line mapping line (step size ~0.16 µm). **b,** The intensity mapping of the 50 Raman spectra. The corresponding color map is on the right of the figure. **c,** The normalized intensity profile of the J_1 mode (red) and E_{2g} mode (black). A sharp boundary between 1T and 2H phase can be observed from both the optical microscope image and intensity profile.

Supplementary Note 1: Transfer process

The transfer process started from spin-coating a layer of polymethyl methacrylate (PMMA) on the substrate to protect the sample (Supplementary Fig. 2a). After heating at 120 °C for 1 min to solidify the PMMA, an adhesive tape with a window was used to cover the PMMA while exposuring the sample area (Supplementary Fig. 2c). The SiO_2/Si was then removed by potassium hydroxide (KOH) etching (Supplementary Fig. 2d); the surrounding tape was used to pull up the sample-PMMA layer and washed in deionized water several times. The sample was dried in ambient environment and put upside-down on a polydimethylsiloxane (PDMS) elastomer (Supplementary Fig. 2 e and f). Finally, the transfer process was performed by target transfer methods under an optical microscope (Supplementary Fig. 2g)[1, 2] and the PMMA can be removed by hot acetone. Annealing at 300 °C in argon or vacuum helps to remove the residues and improve the van der Waals interface.

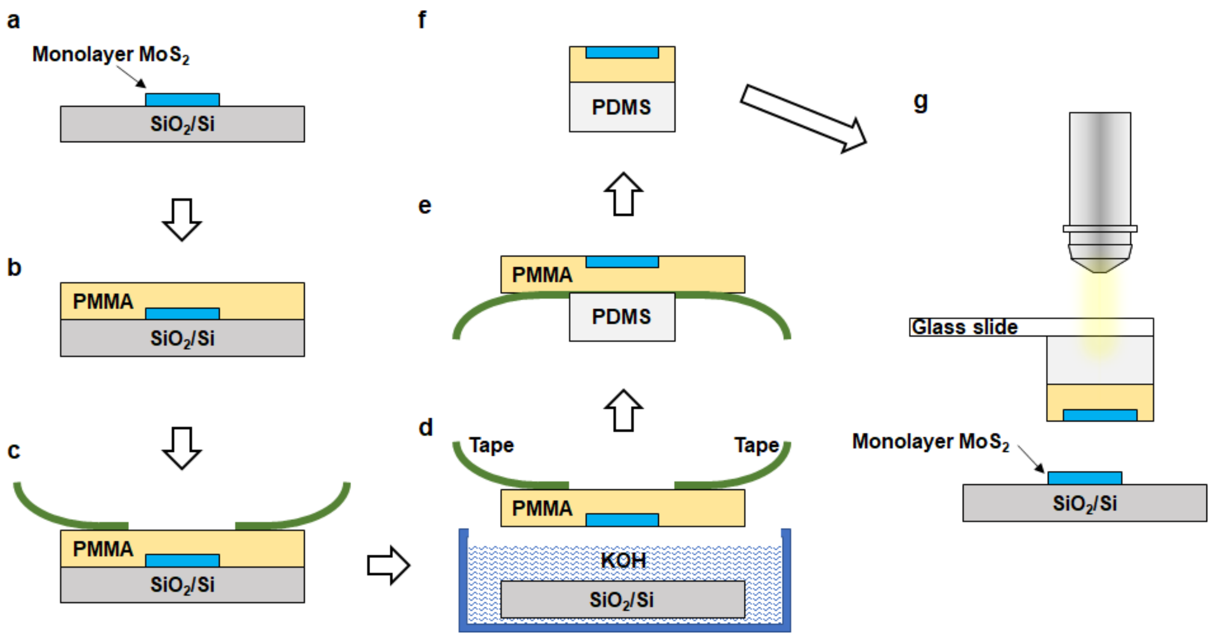

Supplementary Figure 2. Schematic illustration of the transfer process. **a,** CVD grown MoS_2 on SiO_2/Si substrate. **b,** A layer of PMMA is coated on the surface. **c,** An adhesive tape with a window is used to cover the PMMA while exposing the sample area. **d,** The SiO_2/Si substrate is removed by potassium hydroxide (KOH) etching, and the sample is washed in DI water several times. **e,** The dried sample is attached to a PDMS elastomer. **f,** The surrounding tape is removed. **g,** Transferring the sample under an optical microscope.

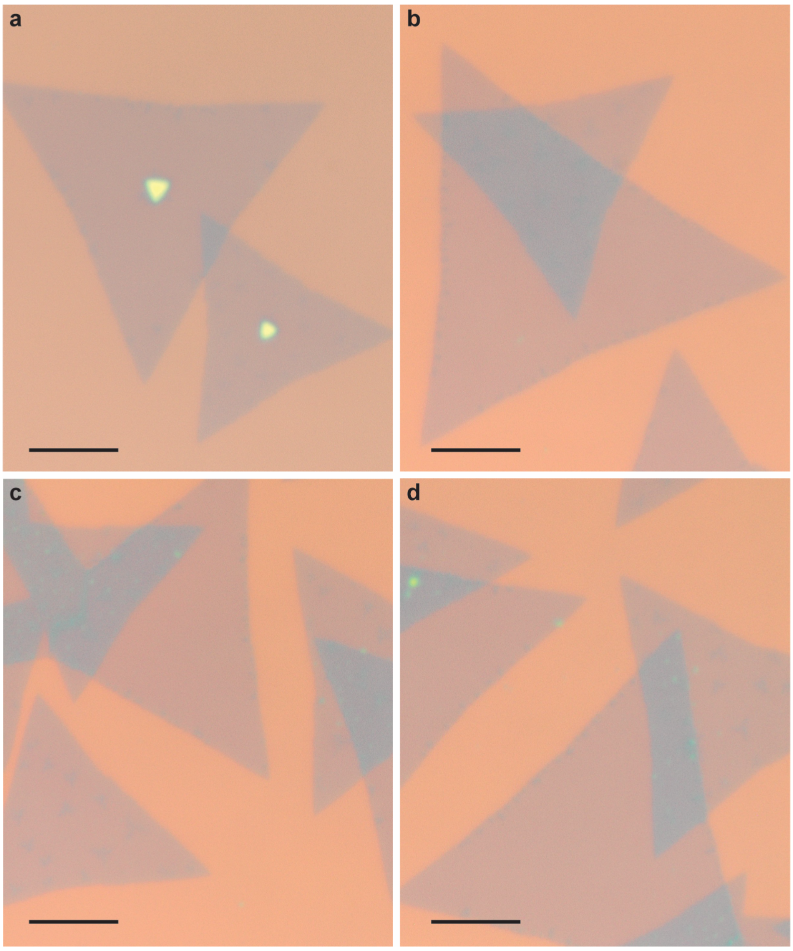

Supplementary Figure 3. Optical microscope images of twisted bilayer MoS_2 with various angles on a substrate in different areas. **a,** area 1. **b,** area 2. **c**, area 3. **d**, area 4. (All scale bars: 10 μm)

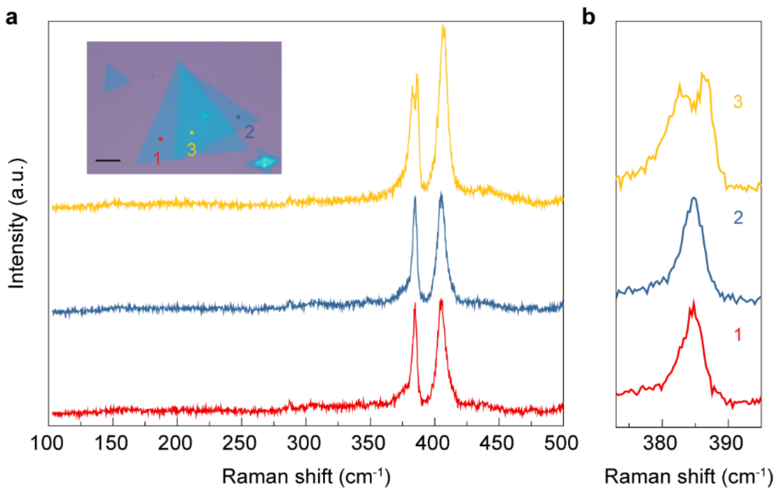

Supplementary Figure 4. a, Raman spectra of the positions 1, 2, and 3 after intercalation. Insert: Optical microscope image of the sample prepared by two dry-transfer processes (scale bar: 20 μm). **b**, Zoom-in image of the E_{2g} peaks after intercalation. The sample was prepared by transferring two monolayer flakes on a new substrate using the transfer method without etching process[3] to minimize surface defects and ensure that every layer experiences the same processing steps.

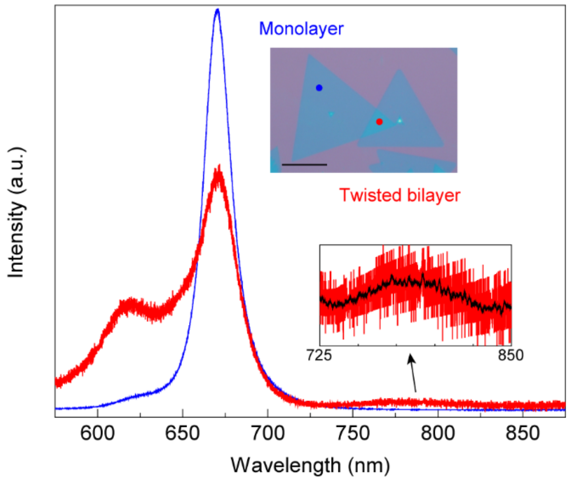

Supplementary Figure 5. The PL spectra of the monolayer (blue) and bilayer (red) MoS_2, and the enlarged diagram of the spectrum between 725 nm and 850 nm, indicating the indirect bandgap of the twisted bilayer MoS_2. Insert: the optical microscope image of the sample (scale bar: 50 μm). The sample with ~ 20° twist angle shows an indirect bandgap of ~1.58 eV (784 nm), which is consistent with literature report.[4]

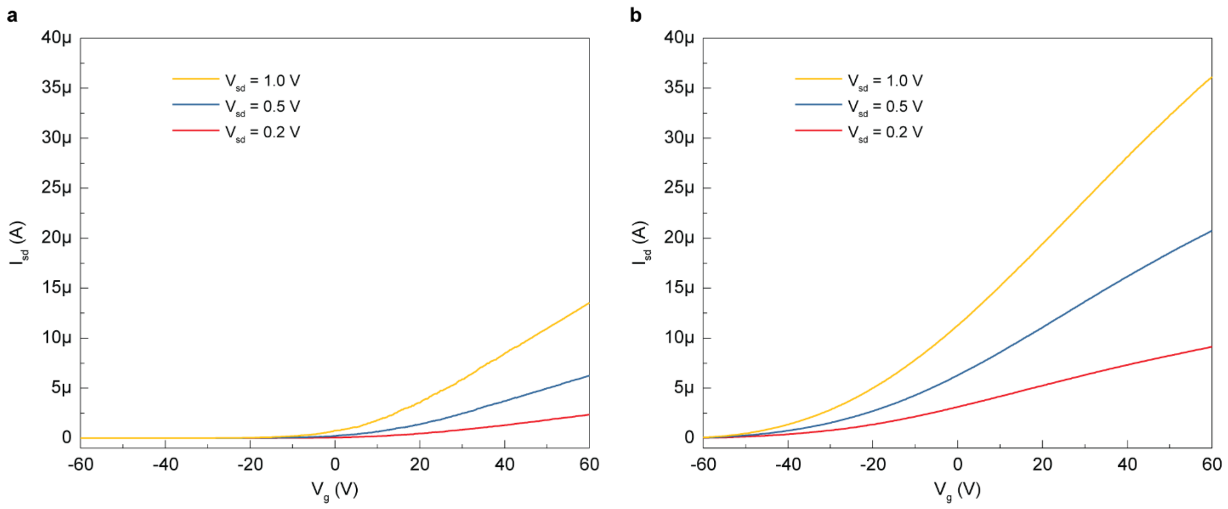

Supplementary Figure 6. a, I_{sd} – V_g characteristics of a pristine twisted bilayer MoS_2 channel at V_{sd} = 1.0 V, 0.5 V, and 0.2 V. **b**, I_{sd} – V_g characteristics of a twisted bilayer MoS_2 channel in the intermediate state at V_{sd} = 1.0 V, 0.5 V, and 0.2 V.

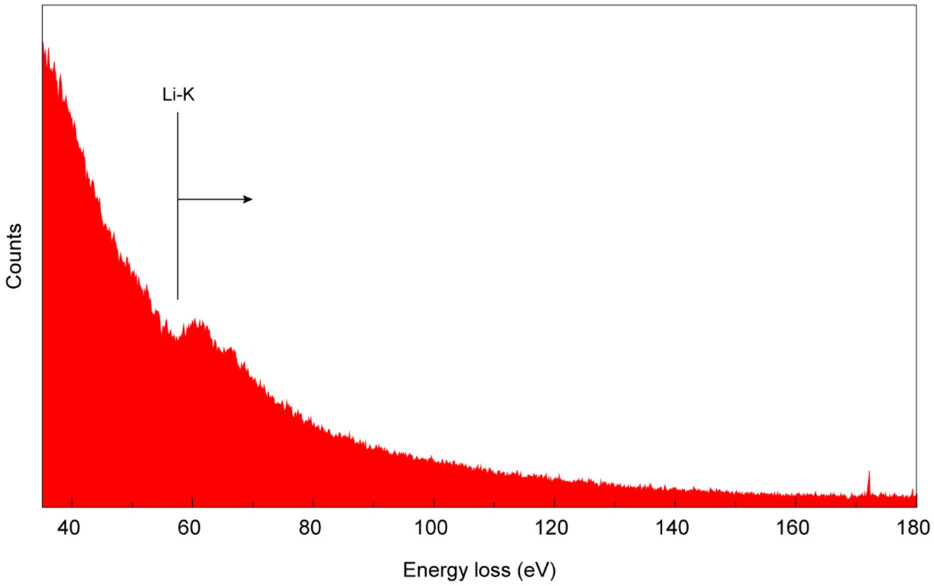

Supplementary Figure 7. Electron energy loss EEL spectra acquired from a twisted bilayer MoS_2 area after 15 minutes of lithium intercalation.

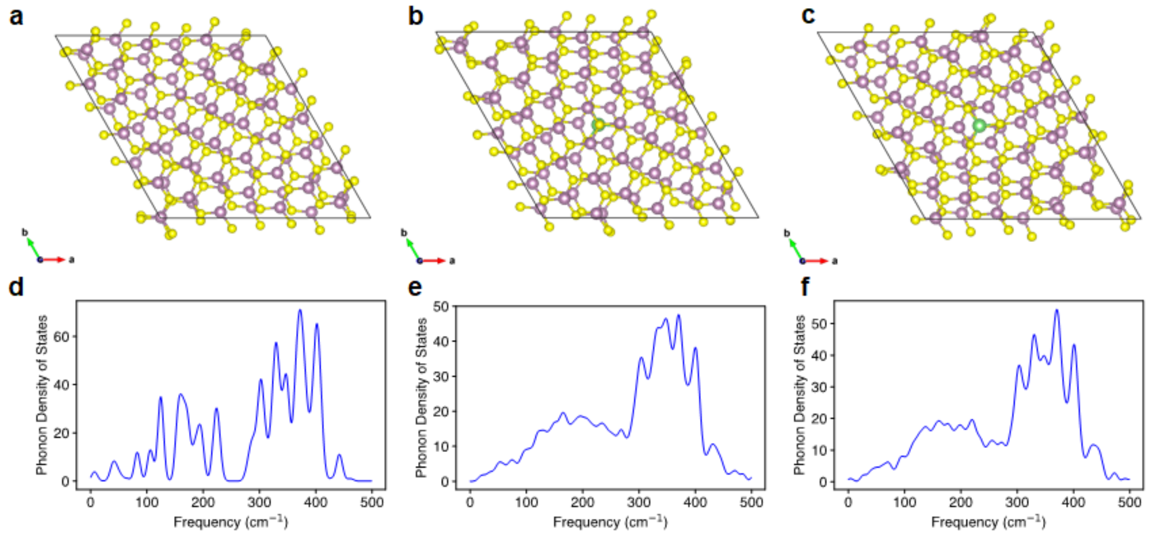

Supplementary Figure 8. a, Schematic illustration of a twisted bilayer MoS_2 with (3,4)-configuration of 9.43° twist angle and supercell lattice constant of 19.3 Å. **b** and **c,** Schematic illustration of the twisted bilayer MoS_2 in (**a**) with one lithium atom in the MX and MM position. **d-f,** The calculated corresponding phonon density of states of the supercells in **a-c**.

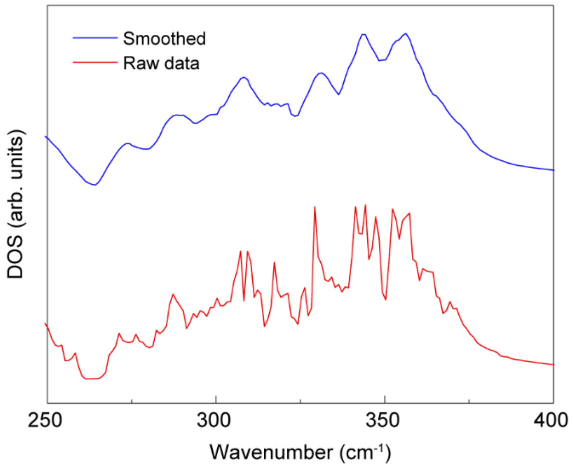

Supplementary Figure 9. The raw data of DFT calculated Li-2H-MoS_2 phonon DOS and the smoothed curve after 15-point Savitzky-Golay filter smoothing.

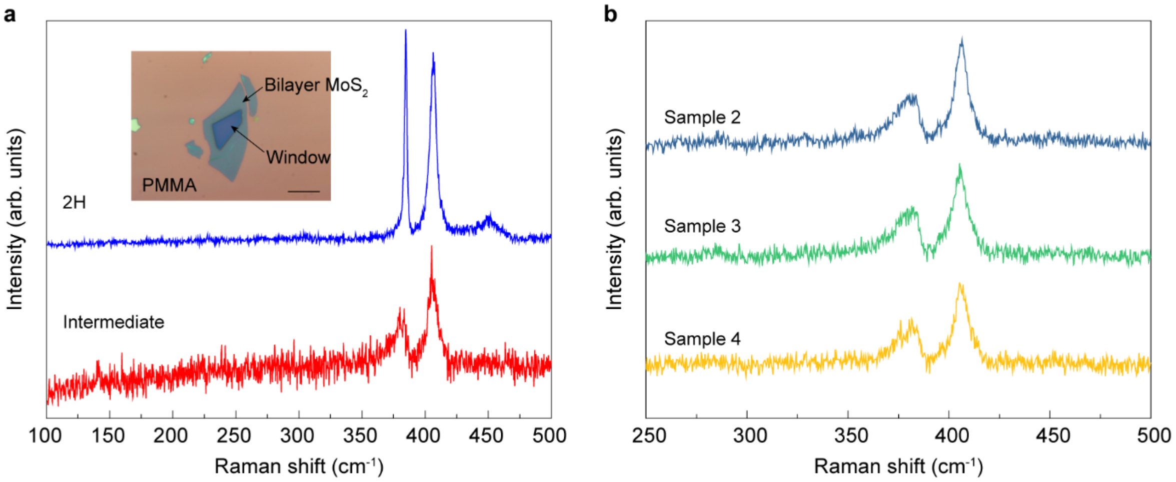

Supplementary Figure 10. a, The Raman spectra of exfoliated bilayer MoS_2 and the intermediate state after ~12 h intercalation. Inset: the optical microscopy image of the exfoliated samples with PMMA covering the edges (scale bar: 10 μm). **b**, The Raman spectra of other exfoliated bilayer MoS_2 samples after ~12 h intercalation. The peak splitting behavior cannot be clearly identified in some samples (**b**), which is probably due the variations of the intercalation time required for the peak splitting behavior. However, the broadening of the E_{2g} peak is affirmed, which is consistent with our conclusion that the lithium intercalation introduces a symmetry breaking in the bilayer MoS_2.

Supplementary References

1. Frisenda, R. et al. Recent progress in the assembly of nanodevices and van der Waals heterostructures by deterministic placement of 2D materials. *Chem. Soc. Rev.* **47**, 53-68 (2018).
2. Huang, S.X. et al. Probing the Interlayer Coupling of Twisted Bilayer MoS_2 Using Photoluminescence Spectroscopy. *Nano Lett.* **14**, 5500-5508 (2014).
3. Liao, M.Z. et al. Precise control of the interlayer twist angle in large scale MoS_2 homostructures. *Nat. Commun.* **11**, 2153 (2020).
4. Liu, K.H. et al. Evolution of interlayer coupling in twisted molybdenum disulfide bilayers. *Nat. Commun.* **5**, 4966 (2014).
