# Supplementary material for: Observation of an intermediate state during lithium intercalation of twisted bilayer MoS2
Source: Nat Commun. 2022 May 30;13:3008. doi: 10.1038/s41467-022-30516-z (PMC9151788; doi:10.1038/s41467-022-30516-z)
Supplement: Supplementary file 2 — Description of Additional Supplementary Files [file 41467_2022_30516_MOESM2_ESM.docx]

File Name: Supplementary Movie 1
Description: The schematic atomic vibrational movements associated with the *E*_2g_ mode before and after intercalation.
